# Health seeking behavior and its determinants for cervical cancer among women of childbearing age in Hossana Town, Hadiya zone, Southern Ethiopia: community based cross sectional study

**DOI:** 10.1186/s12885-018-4203-2

**Published:** 2018-03-16

**Authors:** Yitagesu Habtu, Samuel Yohannes, Tariku Laelago

**Affiliations:** 1 Department of health information technology, Hossana College of health science, Po. Box 159, Hossana, Ethiopia; 2 Department of midwifery, Hossana College of health sciences, Hossana, Ethiopia

**Keywords:** Cervical cancer, Health seeking behavior, Screening

## Abstract

**Background:**

Cervical cancer is one of the most easily preventable forms of female cancers if early screening and diagnosis is made. Low awareness level about the disease and risk factors, beliefs about the disease, poor access to preventive services, affordability of the service and current health service system can influence decision to seek health care services for cervical cancer. The objective of this study was to determine health seeking behaviour and determinant factors for cervical cancer in Hossana town.

**Methods:**

Our study was carried out in Hossana town using community based cross-sectional study design. The study population was women of childbearing age (15–49 years) who had the chance of being randomly selected from the source population. Five hundred ninety five women of childbearing age were included in the study. Systematic random sampling technique was employed to select the study units. Structured and pretested questionnaire was used to collect the data. The collected data were cleaned and entered by EPI info version 3.5.4 and analysed by SPSS version 16. We considered *P*-value < 0.05 to decide statistically significant association between the independent and dependent variables.

**Results:**

The prevalence of health seeking behaviour for cervical cancer among the study participants was only 14.2%. Respondents’ poor knowledge [AOR: 7.25, 95% CI: (1.87, 28.08)], not ever received information [AOR: 52.03, 95% CI: (13.77, 196.52)] and not actively searching information about cervical cancer [AOR: 14.23, (95%CI: (3.49, 57.95)] were significantly associated factors with not seeking health for prevention and control of cervical cancer.

**Conclusion:**

The prevalence of health seeking behaviour for cervical cancer is low. Respondent’ poor knowledge, not ever received information, and not actively searching information about cervical cancer are significantly associated with not seeking health for cervical cancer prevention and control. This study stressed the importance of increasing knowledge, promoting active search of health information and experiences of receiving information from different sources regarding health seeking behaviour.

**Electronic supplementary material:**

The online version of this article (10.1186/s12885-018-4203-2) contains supplementary material, which is available to authorized users.

## Background

Cancer is progressively becoming prominent health threat in high and low income nations among the chronic health problems [1]. It has been exerting negative consequences on the health, economic and social conditions. It is a worldwide problem affecting people in both wealthy and poor countries. Nowadays its prevalence is becoming increasingly high in both low and middle-income countries, where facilities for the control of the disease are inadequate [2].

There are more than 100 types of cancers including breast cancer, skin cancer, lung cancer, colon cancer, prostate cancer, and cervical cancer. Cervical cancer is the leading viral infection of the female reproduction system caused by the Human Papilloma Virus (HPV). Every woman who has been engaged in sexual activity will potentially acquire the virus at a certain time in their life. Repeated infection will also be possible among some sexually active women. Cervical cancer is the second most common cancer among women worldwide. Yet, because of poor health seeking behavior, poor access to screening and treatment services, the majority of deaths occur in women living in low and middle-income nations. Every year, more than 270, 000 women die from cervical cancer, and more than 85% of these deaths are in low and middle income countries [3, 4].

According to population projections, global estimates of cervical cancer are expected to grow to 720,415 incidences and 394,905 deaths in 2025. A remarkable rise has been forecasted in GAVI-eligible countries, with estimated proportion of 58% incidence and 63% in the magnitude of associated mortality [5].

About 35 incident cases of cervical cancer are identified per 100,000 women yearly, and 22.5 per 100,000 women die from the disease in the sub-Saharan African region. This estimate is bigger as compared to 6.6 and 2.5 per 100,000 women, respectively, in North America. These drastic variations could be due to inaccessibility of screening services which hinders early detection and treatment associated with low preventive health seeking behaviour [6].

Cervical cancer has become a double burden among Ethiopian women. Consequently, the government has designed a strategic frame work for its central pillar of improving the health of the population through health promotion, preventive, curative and rehabilitative health services in the health sector. Though, countrywide demonstrative data do not present, the international agency for research on cancer (IARC) projected that cervical cancer is the second most common cause of illness and mortality in 2012. Similarly, data from population based cancer registry in Addis Ababa showed cervical cancer as the second leading cancer which comprised 10.8% of all cancer cases [7].

According to the 2009 World Health Organization report, the age-adjusted incidence rate of cervical cancer in Ethiopia was 35.9 per 100,000 patients with 7619 annual number of incident cases and 6081 deaths yearly. In 2010, an estimated 4648 incident cases and 3235 deaths occurred due to cervical cancer [8, 9].

Although reproductive health in general has been an important area of focus in the country, efforts to address specific area of reproductive tract cancers have been minimal to date. In response to this gap and to the ever-increasing rates of cervical cancer, informed by the success of small pilot projects in generating demand for such services, the federal ministry of health (FMOH) decided (in 2013) to embark on development and implementation of a national cervical cancer prevention and control program. The FMOH national guidelines for cervical cancer prevention and control provide the most current and up-to-date knowledge and direction on cervical cancer screening, treatment and management. These guidelines provide a solid foundation from which service providers in all health facilities, in the public and private sectors including non-governmental organizations, can provide quality and standardized cervical cancer prevention and treatment services [8].

Cervical cancer is one of the most easily preventable forms of female cancers if early screening and diagnosis is made. Low awareness level about the disease and its risk factors, beliefs about the disease, poor access to preventive services, unaffordability of the service and current health service system can all affect decision to seek health care services for cervical cancer [10].

According to sociology literature, health care seeking behavior will be influenced by the individual knowledge, disease perception, socio demographic factors and the availability and accessibility of health services. Depending on these determinants and their interactions, health care seeking behavior is a complex outcome of many factors operating at individual, family, and community level [11].

In Ethiopia, there is inadequate evidence concerning health seeking behavior and associated factors for the control and prevention of cervical cancer at grass root level as nationally indicated in the strategic plan of the prevention and control of chronic diseases [7]. As to the researcher’s knowledge, there are no community based studies conducted so far on cervical cancer screening among the target population except the availability of few facility based studies. Therefore, the objective of this study was to assess health seeking behaviour and the determinant factors for cervical cancer in Hossana town.

## Methods

### Study area and period

Our study was carried out in Hossana town, the administrative principal of Hadiya Zone, South nation’s nationalities and people regional state of Ethiopia in June, 2015. It is 232 and 194 KMs far away from Addis Ababa and the regional capital Hawassa, respectively. There is one Zonal hospital, three health centres and more than 25 clinics in the town. In 2014, the estimated number of residents in the town was 101,849, of which women of childbearing age (WCBA) constitutes 23,731. There were 20,785 households in the study area.

According to the reports of Zonal health department, there were no targeted actions yet concerning non-infectious diseases prevention and control including cervical cancer. Health education and information dissemination program carried out by the health care facilities were majorly addressing communicable diseases. This is despite the fact that non-communicable diseases including cervical cancer are a double burden in the area. Although, the HPV vaccine is generally available and the demand for the vaccine is evident among the target group, the service is currently non-existent in the study setting.

### Study design

We used community based cross-sectional study design.

### Study participants

All WCBA were the source population for our study. Those women who were residents of the town but living for less than six months duration were excluded from the study. Even though, the age group 15–21 have not been routinely accessing cervical cancer screening services, they are included in current study with the fact that some indications for the screening of cervical cancer can be applied for them. The following are some indications that can be applied for any women of child bearing age: women who have had abnormal bleeding like bleeding after intercourse or other abnormal symptoms and women who have been found to have abnormalities on their cervix. Temporary mild cervical cell changes caused by transient HPV infections are also common in women under 21 years [8, 12]. And because this study focuses on the intention to be screened for the disease, participants in the age range of 15–21 years were considered to be potentially at risk and would be important for intervention. Women who have already screened for cervical cancer were also included in the study.

### Sampling

#### Sample size

We used single population proportion formula to calculate the required sample size for the study by using 95% confidence level (CI), Z (1-ά/2) = 1.96), an expected proportion of health seeking behaviour of 50% and, 5% margin of error. As to our knowledge, in the country, there were no previously conducted studies which determined the proportion of health seeking behaviour. Thus, 50% was taken to estimate the required sample size. Moreover, we went more than one sampling stage to reach to the final study units in the process. Consequently, we multiplied our sample size with the design effect of 1.5 to increase the sample size and minimize the variability. Considering the aforementioned assumptions, the sample size was determined as:

$$ \mathrm{n}=\frac{\mathrm{Z}\frac{{\mathrm{a}}^2}{2}\mathrm{P}\left(1-\mathrm{P}\right)}{{\mathrm{d}}^2} $$

$$ \mathrm{n}=\frac{(1.96)^20.5\left(1-0.5\right)}{(0.05)^2}=384(1.5)=576 $$

We included 5% none-response rate in the determination of the sample size needed for the study. Therefore, our final sample size was **595** WCBA.

### Sampling techniques

Systematic random sampling technique was employed to select the study subjects. Initially, all the kebeles (the smallest administrative units in the country) were taken to form a frame. Then, we took five Kebeles by lottery method. The number of households with eligible participants was computed proportionally depending on the number of the study participants in each kebele. We determined the k^th^ value for each selected kebele to decide the sampling interval. We demonstrated spinning pen method at the midpoint of each kebele to choose the track and start of the data collection. The data collection activity was continued in the chosen direction until the sample size for that particular kebele has been reached. We employed a lottery method to choose one study participant in a case when more than one women present in the selected household.

### Data collection and measurement

We adapted a questionnaire from similar publications and modified in line with the objectives of the study in order to comply with the local setting (Additional file 1). The instrument was structured and tested in 5% of our sample size before the actual data collection. Firstly, we prepared the instrument in English and translated into Amharic language then, re-translated back to English. Ten female diploma holders graduated in health fields collected the data through face to face interview. The supervisors checked the consistency and completeness of the filled questionnaire on a daily bases.

Two days training was provided for the data collectors and supervisors on the techniques how to collect the data and supervisory skills. Supervision was carried out on a daily bases for the whole duration of the data collection process.

The socio-demographic factors included were age, education status, marital status, occupation, occupation of the respondent, and educational status of husband, household income, parity, religion, and ethnicity.

Data were also collected on behavioral factors including perceived susceptibility, perceived severity, perceived benefit, sexual practice, multiple sexual partnerships, use of condoms and cigarette smoking. Moreover, data were collected on health service factors including availability of and access to the service, active searching of health information, information education, and communication regarding prevention of cervical cancer.

Health seeking behavior for this study was the intention of WCBA to be screened for cervical cancer before they develop signs and symptoms of the disease or the preventive health behaviours of the disease experienced by the participants. Ten structured knowledge assessing questions and twenty five sub classified items were prepared to determine the knowledge level of the participants about the disease. Those who have scored the third quartile value and below were considered to be having poor knowledge score and those who have scored above the third quartile value were taken to be having good knowledge score. The study participants were asked whether they know about Pap smear test or not. It was also used to assess the knowledge level of women.

### Data processing and analysis

The data were first cleaned and entered by EPI info version 3.5.4. The data were transported to SPSS version 16 for statistical analysis. We used descriptive data analysis technique such as frequency tables, graphs and summary measures for the study variables. Logistic regression analysis was employed to identify associated factors for not seeking health regarding prevention of cervical cancer. We used odds ratio at 95% CI to show the strength of the association between health seeking behaviour and the independent variables. Those variables which showed statistically significant association with not seeking health for cervical cancer, in bivariate analysis, were taken to multivariate logistic regression model to control the confounding effects of independent variables. We used *P*-value < 0.05 to determine the presence of statistically significant relation between the independent and outcome variables.

## Results

### Socio-economic and demographic features of the study participants

The actual size of the respondents in our research was 583 with the response rate of 98%. The minimum and maximum age of the participants was 18 and 48 years, respectively. The median age of the study subjects was 28 years with standard deviation of ± 6.83. Majority of the study subjects, 366 (62.8%), are currently married. Protestant religion constituted 388 (66.6%). Adventist, Catholic, Jehovah, and Apostolic altogether constituted 12.3%. Hadiya 368 (63.1%) and Kembata 79 (13.6%) were the two dominant ethnic groups. About 248 (42.5%) respondents were housewives (Table 1).

**Table 1:** Socio-economic and demographic features of the study participants, Hossana town, Ethiopia, June, 2015

Back ground variable	Categories	Frequency	%
Current Marital status (*n* = 583)	Married	366	62.8
	Single	149	25.6
	Widowed	26	4.5
	Separated	25	4.3
	Divorced	17	2.9
Religion (n = 583)	Protestant	388	66.6
	Orthodox	123	21.1
	Islam	33	5.7
	Others	39	6.6
Ethnicity (n = 583)	Hadiya	368	63.1
	Kembata	79	13.6
	Amhara	57	9.8
	Gurage	41	7.0
	Others^a^	38	6.6
Respondents’ age(n = 583)	<=23	135	23.2
	24–27	128	22
	28–34	170	29.2
	> = 35	150	25.6
Respondents’ education (n = 583)	No education	74	12.7
	Primary education (1–8)	172	29.5
	Secondary education (9–12)	177	30.4
	Tertiary education (12 plus)	160	27.4
Respondents occupation (n = 583)	House wife	248	42.5
	Employee	127	21.8
	Student	85	14.6
	Merchant	71	12.2
	Others^b^	52	8.9
Parity(n = 583)	0	162	27.8
	1	82	14.1
	2–4	238	40.8
	> = 5	101	17.3
Husband’s occupation(*n* = 389)	Employee	155	26.6
	Merchant	140	24
	Daily worker	30	5.1
	Farmer	21	3.6
	Others^c^	43	7.3
Husband’s education (n = 389)	No education	14	2.4
	Primary education (1–8)	98	16.8
	Secondary education (9–12)	122	20.9
	Tertiary education (12 plus)	155	26.6
Monthly income in USD(n = 583)	< 72	134	23.0
	72–143	212	36.4
	144–215	97	16.6
	> 215	140	24.0

Others^a^Silte, Wolayita, Gamo,^b^daily labourer, house maid, farmers, ^c^daily labourer, cattle feeders

The income distribution showed that, 179.38 ± 209.10 USD (United States Dollar) was the average monthly income of the respondents’ families.

Two hundred twelve, (36.4%) of the respondents’ families have their monthly income between 71.43 and 142.86 USD. Majority, 238 (40.8%) of the study subjects were multi-paras with the average parity of 2.3 ± 2.2SD (Table 1).

### Health seeking behavior

The proportion of health seeking behavior for cervical cancer among the study participants was only 83 (14.2%). Of which, 76 (91.6%), of the participants who showed health seeking behavior did so because other people recommended the service for them before the survey. The service was suggested for 34(44.6%) by health workers for 17 (22.4%) by neighbours, for 10 (13.2%) by workmates, for 8 (10.5%) by partner and for 7 (9.2%) by relatives. Among those participants who had showed health seeking behavior, 58 (69.9%) of them reported that they had already been screened for the cervical cancer. The reasons for not being screened for cervical cancer were unavailability of the service nearby, 20 (3.4%), not being informed of where to get the service, 12 (2.1%), economic problem, 3 (0.5%), fear of discrimination, 2 (0.3%) and others 5(0.9%).

Nearly nine out of ten study participants (85.8%) had not yet intended to be screened for cervical cancer due to different reasons. Among those participants who did not show health seeking behavior, more than one third, 209 (35.8%) of the participants’ major reason for not seeking health for cervical cancer is not having heard about the disease followed by never had the illness before, 111 (19%) (Table 2).

**Table 2:** Major reasons for not seeking health for cervical cancer, Hossana town, Ethiopia, June 2015

Major reasons cited for not seeking health	Frequency	Percent
I have not heard about the disease	209	35.8
I never had illness	111	19.0
I felt that the disease is not serious	76	13.0
I am not aware of screening test	52	8.9
Service is not available nearby	32	5.5
Other reasons	20	3.3
Total	500	85.6

### Knowledge of women on cervical cancer

**Fig. 1:**
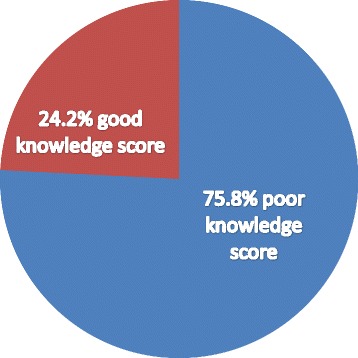
The level of knowledge about cervical cancer for health seeking behavior in Hossana town, Ethiopia, June 2015

A twenty five item composite score of the knowledge was used to measure the knowledge level of respondents regarding vulnerability, risk factors, signs and symptoms and prevention methods of cervical cancer. The cumulative score of knowledge of participants about cervical cancer was estimated using the quartiles. Accordingly, 142 (24.2%) of the respondents scored above the third quartile value in knowledge questions, whom are considered to be having good knowledge score (Fig. 1).

### Health care related factors

Only, 95 (16.3%) of the respondents had ever received information from health professionals regarding cervical cancer. The two major information sources for cervical cancer were government (11.5%) and private health institutions (3.4%).

Eighty five (14.6%) of the respondents have been purposefully seeking cervical cancer related information from different sources before the study. Among these, TV (11.5%), health workers (4.1%), radio (3.9%), internet (3.6%), magazine (3.3%), and newspaper (1.5%) were reported information sources actively searched by the study participants.

### Factors associated with health seeking behavior of cervical cancer

From the multivariate logistic regression analysis, poor knowledge score, not receiving information and not having active search of health information showed a significant association with not showing health seeking behavior for prevention and control of cervical cancer when adjusted for all other variables. Individuals having poor knowledge score were about 7 times more likely not to show health seeking behavior when compared to those who have had good knowledge score for prevention and control of cervical cancer [AOR: 7.25, 95% CI: (1.87, 28.08)]. Similarly, those who had never received information about cervical cancer were about 52 times more likely for not showing health seeking behavior when compared to those who had ever received information from any sources, even if the precision is very low with wide confidence interval [AOR: 52.04 %CI: (13.77, 196.52)]. Likewise, participants who were not actively seeking health information about cervical cancer were 14 times more likely for not having health seeking behaviour as compared to those who were actively searching health information about cervical cancer [AOR: 14.23, (95%CI: (3.49, 57.95)] (Table 3).

**Table 3:** Factors associated with health seeking behavior for cervical cancer in Hossana town, Ethiopia, June 2015

Variable	Variable category	frequency	Crude ORb 95% CI	Adjusted OR 95% CI	*P* value
Respondent age	≤ 23	135	3.49 (1.52, 7.97)	1.74(0.21,14.13)	0.60
	24–27	128	1.43 (0.74, 2.77)	2.00(0.38,10.51)	0.41
	28–34	170	0.98 (0.56, 1.74)	2.70(0.57,12.63)	0.21
	≥ 35	150	1		0.00
Religion	Orthodox	123	1		0.00
	Protestant	388	0.81 (0.43, 1.52)	1.99(0.34,11.73)	0.45
	Islam	33	0.93 (0.29, 3.04)	0.12(0.01,1.72)	0.12
	Others	39	0.29 (0.12, 0.69)	0.28(0.02,3.49)	0.32
Respondent’s education	No education	74	37.19 (5.03, 274.89)	14.64(0.66,322.67)	0.08
	Primary	172	8.25 (4.03, 16.92)	1.72(0.32,9.32)	0.53
	Secondary	177	4.50 (2.50, 8.10)	7.59(1.45,39.70)	0.02
	Tertiary	160	1		0.00
Husband’s occupation	Employee	155	1		0.00
	Merchant	140	2.52 (1.33, 4.76)	3.19(0.78,12.93)	0.10
	Farmer	21	6.50 (0.84, 50.01)	2.30(0.03,159.92)	0.70
	Daily worker	30	9.42 (1.24, 71.49)	3.66(0.18,74.25)	0.39
	Others	43	3.17 (1.06, 9.44)	10.85(0.89,131.27)	0.06
Knowledge score^a^	Poor	442	22.00 (12.23, 39.56)	7.25(1.87,28.08)	0.004
	Good	141	1		0.001
Ever received information^a^	Yes	95	1		
	No	488	79.42 (40.22, 156.83)	52.03(13.77,196.52)	0.001
Active health information seeking^a^	Yes	85	1		
	No	498	40.64 (22.12, 74.69)	14.23(3.49,57.95)	0.001

^a^significantly associated factors

## Discussion

Health seeking behavior for dual application of primary and secondary prevention strategies offers an opportunity for comprehensive prevention and control strategies of cervical cancer. Unless the level of intention to be screened for cervical cancers is increased, prevention and control of the disease is more difficult and challenging. Hence, this study determined the extent of health seeking behaviour and its associated factors for cervical cancer prevention and control among WCBA.

In current study 14.2% of women had health seeking behaviour to be screened for cervical cancer. This is less than what was observed in the study of Malaysia and Uganda, which showed about 56% and 63 of women had intention to be screened, respectively [13, 14].

Among women who had health seeking behaviour for cervical cancer, 30% did not get screening service. The reasons for not being screened for cervical cancer were unavailability of the service nearby, not being informed of where to get the service, and economic problem. The mentioned reasons were also indicated in many studies [13, 15–17].

As shown in the descriptive analysis, those women who did not have preventive health seeking behavior reported that majority of them had not ever heard about the disease. This is followed by not having had illness related to the diseases before. This finding showed that participants had much lower awareness level in this study when compared to findings of other studies done in Ethiopia [18] and other countries [19–23]. This low level of awareness could be attributed to low attention given by the media, variations in the involvement of cervical cancer education in curricula and its exposure. Moreover, differences in socio-cultural conditions, health education at healthcare facilities and other behavioral change interventions regarding the prevention and control of cervical cancer might have contributed for the low awareness level.

The current study showed that poor knowledge score, not experiencing active information search about cervical cancer and not receiving information from any sources were significantly associated with not showing health seeking behavior for prevention and control of the disease. These results have been supported by findings of other research [24].

### Limitation of the study

The contributions of the spouses in the intention to seek health behaviour were not included in the study.

The limited availability or access to clinic services that may be offering Pap smear screening test.

## Conclusion

The prevalence of health seeking behaviour for cervical cancer is low. Respondents’ poor knowledge, not ever received information and not actively searching information about cervical cancer were significantly associated with not seeking health for cervical cancer prevention and control.

Increasing knowledge on cervical cancer, encouragement of active search of information and experience of receiving information from relevant source about cervical cancer should be promoted. Incorporation of cervical cancer prevention and control strategy with maternal health care services is essential at all levels of health care. Designing and institutionalizing new strategies that encourage screening practices should be part of the role of health care providers, researchers and stakeholders. Finally, additional research is required to clearly comprehend the nature of factors operating against/for health seeking behaviour for cervical cancer among WCBA.

## Additional file

Additional file 1: Questionnaire, English version. The questionnaire uploaded as Additional file 1 was used to assess health seeking behaviour and its determinants for cervical cancer among women of child bearing age in Hossana town, Hadiya zone, Southern, Ethiopia. (PDF 212 kb)

## Abbreviations

ETB: Ethiopian Birr
FMOH: Federal minster of health
GAVI: Global alliance for vaccination and immunization
HPV: Human papilloma virus
KM: Kilometre
TV: Television
USD: United states dollar
WCBA: Women of childbearing age

## References

[1] World Health Organization. Attaining the nine global non-communicable diseases targets; a shared responsibility. Global status report on non-communicable diseases. Geneva: WHO; 2014.

[2] Ferlay J, Soerjomataram I, Ervik M, Dikshit R, Eser S, Mathers C, et al. GLOBOCAN 2012 v1.0, Cancer incidence and mortality worldwide: IARC Cancer Base no. 11. Lyon, France: International Agency for Research on Cancer, 2013. (Available from: http://globocan.iarc.fr).

[3] WHO. Comprehensive cervical cancer prevention and control: A healthier future for girls and women, Geneva, Switzerland, 2013.

[4] WebMD. Cancer health cancer:www.m.webmd.com/cancer-toc.

[5] De Sanjose, Serrano B, Castellsague X, Brotons M, Munoz J, Bruni L et.al. Human papillomavirus (HPV) and related cancers in the global alliance for vaccines and immunization (GAVI) Countries: A WHO/ICO HPV Information Centre Report; 2012. 10.1016/S0264-410X(12)01435-1 23510764

[6] WHO. Latest world cancer statistics, Release, 2012.

[7] FMOH: National Strategic Action Plan (NSAP) for prevention & control of non-communicable diseases in Ethiopia from 2014-2016.

[8] Federal Democratic Republic of Ethiopia, Ministry of Health .Guideline for Cervical Cancer Prevention and Control in Ethiopia, FMOH ,Addis ababa, 2015.

[9] WHO/ICO: Human papilloma virus and related cancers in Ethiopia. In Summary report; 2009.

[10] Yu FQ, Murugiah MK, Khan AH, Mehmood T (2015). Meta-synthesis exploring barriers to health seeking behaviour among Malaysian breast Cancer patients. Asian Pac J Cancer Prev.

[11] Chadza E, Chirwa E, Maluwa A, Malata A, Kazembe A, Chimwaza A. Factors that contribute to delay in seeking cervical cancer diagnosis and treatment among women in Malawi. J Sci Res Health. 2012:1015–22.

[12] Cervical Cancer Screening Program. Offline manual for health professionals BC cancer agency. Tenth edition, 2013.

[13] Abdullah NN, Al-Kubaisy W., Mokhtar M. Health Behaviour Regarding Cervical Cancer Screening Among Urban Women in Malaysia; Elsevier. Social and behavioural sciences, 2013;85: 110–117.

[14] Twinomujuni C, Nuwaha F, Babirye JN (2015). Understanding the low level of cervical Cancer screening in Masaka Uganda using the ASE model: a community-based survey. PLoS One.

[15] Birhanu Z, Abdissa A, Belachew T, Deribew A, Segni H, Vivien T (2012). Health seeking behavior for cervical cancer in Ethiopia: a qualitative study. Int J Equity Health.

[16] Hailu A, Haile Mariam D. Patient side cost and its predictors for cervical cancer in Ethiopia: a cross sectional hospital based study. BMC Cancer. 2013;13(69) http://www.biomedcentral.com/1471-2407/13/69. 10.1186/1471-2407-13-69 PMC3576296 23391288

[17] Torres E, Erwin DO, Treviño M, Jandorf L. Understanding factors influencing Latina women’s screening behaviour: a qualitative approach. Oxford journals. 2012;28:772–83. 10.1093/her/cys106 PMC3772327 23131588

[18] Gedefaw A, Astatkie A, Tessema GA (2013). The prevalence of precancerous cervical Cancer lesion among HIV-infected women in southern Ethiopia: a cross-sectional study. PLoS One.

[19] Mwaka AD, Med M, Orach CG, Were EM, Lyratzopoulos G, Wabinga H, Roland M, FRCP. Awareness of cervical cancer risk factors and symptoms: cross-sectional community survey in post-conflict northern Uganda. Health Expect. 2015;19:854–67. 10.1111/hex.12382 PMC4957614 26205470

[20] Ekechi C, Olaitan A, Ellis R, Koris R, Amajuoyi A, Marlow LAV. Knowledge of cervical cancer and attendance at cervical cancer screening: a survey of black women in London. BMC Public Health. 2014;14(1096) http://www.biomedcentral.com/1471-2458/14/1096. 10.1186/1471-2458-14-1096 PMC4216339 25339243

[21] Amosu AM, Degun AM, Babalola AO, Thomas MA (2011). Level of specific knowledge, awareness, perception, and screening behavior regarding carcinoma of the cervix among rural women in Iwo local government area, Osun state, Nigeria. Ann Biol Res.

[22] Julinawati S, Cawley D, Domegan C, Brenner M, Rowan NJ (2013). A review of the perceived barriers within the health belief model on pap smear screening as a cervical cancer prevention measure. J Asian Sci Res.

[23] Hoque M, Hoque E, Kader SB. Evaluation of cervical cancer screening program at a rural community of South Africa. East Afr J Public Health. 2008;5(2). 19024420

[24] Getahun G, Mazengia F, Abuhay M, Birhanu Z. Comprehensive knowledge about cervical cancer is low among women in Northwest Ethiopia. BMC Cancer. 2013;13(2) http://www.biomedcentral.com/1471-2407/13/2. 10.1186/1471-2407-13-2 PMC3559275 23282173

