# Supplementary material for: Health seeking behavior and its determinants for cervical cancer among women of childbearing age in Hossana Town, Hadiya zone, Southern Ethiopia: community based cross sectional study
Source: BMC Cancer. 2018 Mar 16;18:298. doi: 10.1186/s12885-018-4203-2 (PMC5857120; doi:10.1186/s12885-018-4203-2)
Supplement: Supplementary file 1 — Questionnaire, English version. The questionnaire uploaded as Additional file 1 was used to assess health seeking behaviour and its determinants for cervical cancer among women of child bearing age in Hossana town, Hadiya zone, Southern, Ethiopia. (PDF 212 kb) [file 12885_2018_4203_MOESM1_ESM.pdf]

Annex: 1. Questionnaire, English version

Information sheet and Consent form

Research Title: Health Seeking Behavior and its determinants for cervical cancer among women of child bearing age in Hossana town, Hadiya zone, SNNPR, Ethiopia

Dear Respondent:

My name is ____________. I am working for Hossana College of Health sciences. Currently I am working with the community to contribute and support implementation of community health activities. Now, I am going to collect information about health care seeking behaviour and related factors for cervical cancer in this area. You are randomly chosen to be included in the study as part of the sample population to complete the questionnaire designed by the researcher. The information obtained in this study will be used only for research purposes. The data you will provide is very helpful to achieve the intended objectives of the study. Any information obtained will be kept strictly confidential and will not be exposed to any other body. Involvement in this study is optional and is only in voluntary basis and you can drop any individual question or the whole questionnaire. But your participation and contribution in the study is very important to come up with important findings which may help local health planners to intervene the problem locally. The questionnaire probably takes between 30-40 minutes to complete.

Do you agree to participate in this study?

Yes, continue No, thank you!

Instructions for interviewer

Introduce yourself and read all the information given on the information sheet and consent form for the interviewee prior to interviewing

After the interviewee understand and show willingness to participate, take the verbal consent to confirm the voluntary participation and to respect the right of respondents

Read the questions for interviewee and circle the response in the option part or on the space provided, if appropriate, by using pen **only**.

Household Identification
**Woreda :_________________Kebele code**: |____|____| household code : |____|____||____| <br>Kebele____Village : <br>____________________________________ <br>Name of data collector _____________________________________ sig.___________ date _________ <br>Name of Supervisor _______________________________________ sig. ____________ date ________ <br>Date of interview (dd/mm/yyyy)|____|____||____|____| <br>Time at beginning of interview _____:______

SECTION-I SOCIO-DEMOGRAPHIC CHARACTERISTICS OF THE RESPONDENTS

S.N	Questions	Response and Coding	Skip
**101**	Age of the respondent in years	____________years	
**102**	Current marital status	1. Married<br>2. Single<br>3. Divorced<br>4. Widowed<br>5. Separated	
**103**	Religion	1. Orthodox<br>2. Protestant<br>3. Muslim<br>4. Catholic<br>5. Others (specify)____________	
**104**	Ethnicity	1. Hadiya<br>2. Kembata<br>3. Guragie<br>4. Siltie<br>5. Amhara<br>6. Others (specify)____________	

**105**	The highest grade completed by the respondent	1. Illiterate<br>2. Able to read and write<br>3. Elementary (1 - 6)<br>4. Junior (7 - 8)<br>5. Secondary (9 - 12)<br>6. Tertiary (12+)	
**106**	Occupation of the respondent	1. Employee (GO/NGO)<br>2. House wife<br>3. Merchant<br>4. Student<br>5. Farmer<br>6. Daily worker<br>7. Others (Specify)__________	
**107**	Occupation of the husband if any	1. Employee (GO/NGO)<br>2. Merchant<br>3. Student<br>4. Farmer<br>5. Daily worker<br>6. Others (Specify)__________	
**108**	Education of the husband if any	1. Illiterate<br>2. Able to read and write<br>3. Elementary (1 - 6)<br>4. Junior (7 - 8)<br>5. Secondary (9 - 12)<br>6. Tertiary (12+)	
**109**	Family monthly income (salary, farming, trade, rental etc)	1. ___________ in ETB	
**110**	Parity of the respondent	__________ births	

SECTION II AWARNESS AND HEALTH SEEKING BEHAVIOUR FOR CERVICAL CANCER

S.N	Questions	Response and Coding	Skip
**201**	Have you had an intention to be screened for cervical cancer in the health facility?	1. Yes<br>2. No	If yes go to 203
**202**	If no for question no 201 what would be your reason?	1. Have not heard about the disease<br>2. I felt that the disease is not serious<br>3. Service is not available nearby<br>4. Not aware of screening test<br>5. Others (specify)______________	
**203**	If yes for question no 201, has anyone ever recommended for you to be tested or screened for cervical cancer?	1. Yes<br>2. No	If no go to 205
**204**	If yes for question No 203, who recommended it for you?	1. Spouse<br>2. Colleague<br>3. Neighbor<br>4. Health worker<br>5. Relatives<br>6. Others (specify)_______	
**205**	If yes for question No 201, have you ever been screened for it?	1. Yes<br>2. No	If yes go to 301
**206**	If no for question no 205 what is the reason for not being screened	1. Service is not available nearby<br>2. Financial problem<br>3. Unaware of where to get the service<br>4. Fear of discrimination<br>5. Others, specify______________	

SECTION III KNOWLEDGE ABOUT THE CERVICAL CANCER

S.N	Questions	Response and Coding	Skip
**301**	What do you think causes cervical cancer?	1. HIV<br>2. HPV<br>3. M. tuberculosis<br>4. Do not know	
**302**	Dou you think that early detection of the cervical cancer is helpful?	1. Yes<br>2. No	
**303**	If yes for question no 302, what do you think is the advantage?	1. To prevent<br>2. To control<br>3. Others specify ________________	
**304**	Do you think that one time screening for cervical cancer is enough?	1. Yes<br>2. No	
**305**	Which group of women do you think should get pap smear?	1. Women with gynecological problems only<br>2. Pregnant women only<br>3. All women of child bearing age<br>4. Sexually active women only<br>5. Do not know	
**306**	Which condition do you think increases the chance of having cervical cancer for women? *(Circle all that apply)*	1. Infection with HPV<br>2. Having a weakened immunity<br>3. Starting to have sex before age 17<br>4. Use of oral contraceptive pills<br>5. Having many children<br>6. Having STI before<br>7. Multiple sexual partners<br>8. Not using condom during sex<br>9. Smoking<br>10. Family history of cervical cancer<br>11. I do not know	
**307**	Which one of the following common signs and symptoms are of cervical	1. Vaginal bleeding<br>2. Vaginal discharge	

	cancer? *(Circle all that apply)*	3. Pain during sex<br>4. Pelvic pain	
**308**	Do you think that cervical cancer is preventable?	1. Yes<br>2. No	If no go to 309
**309**	If yes for question no 307 how do you think is prevented? *(Circle all that apply)*	1. Early detection and treatment<br>2. Vaccination<br>3. Risk reduction<br>4. Do not know	
**310**	Do you think that cervical cancer has cure once it has been diagnosed?	1. Yes<br>2. No	

SECTION IV HEALTH SERVICE RELATED FACTORS

**S.N**	**Questions**	**Response and Coding**	**Skip**
**401**	Have you ever received information from any health provider about cervical cancer?	1. Yes<br>2. No	If no go to 404
**402**	If yes for question no 401, from where did you receive?	1. Government health facility<br>2. Private health facility<br>3. Drug vendors<br>4. Traditional healers<br>5. Others (specify)__________	
**403**	If yes for question no 401, did you get it from the nearby health facility?	1. Yes<br>2. No	
**404**	Have you been looking for the information about cervical cancer?	1. Yes<br>2. No	If no go to 501
**405**	If yes for question number 403, from where have you searched for the information? *(Circle all that apply)*	1. TV<br>2. Radio<br>3. Health professionals<br>4. News paper<br>5. Magazine<br>6. Internet<br>7. Others (specify)________	

SECTION V BEHAVIOURAL FACTORS OF THE RESPONDENTS

S.N	Questions	Response and Coding	Skip
501	I am not aware of cervical cancer, I can't have it.	1. Strongly agree<br>2. Agree<br>3. Neutral<br>4. Disagree<br>5. Strongly Disagree	
502	Cervical cancer is a deadly disease.	1. Strongly agree<br>2. Agree<br>3. Neutral<br>4. Disagree<br>5. Strongly Disagree	
503	Cervical cancer is an infection that can be transmitted sexually.	1. Strongly agree<br>2. Agree<br>3. Neutral<br>4. Disagree<br>5. Strongly Disagree	
504	Cervical cancer has no cure.	1. Strongly agree<br>2. Agree<br>3. Neutral<br>4. Disagree<br>5. Strongly Disagree	
505	Cervical cancer cannot make me infertile.	1. Strongly agree<br>2. Agree<br>3. Neutral<br>4. Disagree<br>5. Strongly Disagree	
506	All female are at risk of having Cervical cancer.	1. Strongly agree<br>2. Agree<br>3. Neutral	

		4. Disagree<br>5. Strongly Disagree	
**507**	I perceive great benefit in going to the clinic regularly for a medical check-up	1. Strongly agree<br>2. Agree<br>3. Neutral<br>4. Disagree<br>5. Strongly Disagree	
